# Autophagy and Apoptosis Act as Partners to Induce Germ Cell Death after Heat Stress in Mice

**DOI:** 10.1371/journal.pone.0041412

**Published:** 2012-07-25

**Authors:** Mianqiu Zhang, Min Jiang, Ye Bi, Hui Zhu, Zuomin Zhou, Jiahao Sha

**Affiliations:** State Key Laboratory of Reproductive Medicine, Department of Histology and Embryology, Nanjing Medical University, Nanjing, Jiangsu Province, China; University of Medicine and Dentistry of New Jersey, United States of America

## Abstract

Testicular heating suppresses spermatogenesis which is marked by germ cell loss via apoptotic pathways. Recently, it is reported that autophagy also can be induced by heat treatment in somatic cells. In this study, the status of autophagy in germ cells after heat treatment, as well as the partnership between autophagy and apoptosis in these cells was investigated. The results demonstrated that besides initiating apoptotic pathways, heat also induced autophagic pathways in germ cells. Exposure of germ cells to hyperthermia resulted in several specific features of the autophagic process, including autophagosome formation and the conversion of LC3-I to LC3-II. Furthermore, the ubiquitin-like protein conjugation system was implicated as being likely responsible for heat-induced autophagy in germ cells since all genes involving this system were found to be expressed in the testes. In addition, the upstream protein in this system, Atg7 (Autophagy-related gene 7), was found to be expressed in all types of spermatogenic cells, and its expression level was positively correlated with the level of autophagy in germ cells. As a result, Atg7 was selected as the investigative target to further analyze the role of autophagy in heat-induced germ cell death. It was shown that down expression of Atg7 protein resulted in the notable decrease in the level of autophagy in heat-treated germ cells, and this down-regulation of autophagy caused by Atg7 knockdown further reduced the apoptotic rate of germ cells. These results suggest that autophagy plays a positive role in the process of germ cell apoptosis after heat treatment. In conclusion, this study demonstrates that heat triggers autophagy and apoptosis in germ cells. These two mechanisms might act as partners, not antagonist, to induce cell death and lead to eventual destruction of spermatogenesis.

## Introduction

Spermatogenesis is a highly ordered process that is dependent on well-balanced germ cell proliferation, differentiation and death in the testes [1], [2]. This process can be disturbed by several factors, including chemical insults, withdrawl of gonadotropin or testosterone, heat, radiation, etc [3]–[7]. Testicular heating suppresses spermatogenesis in several mammalian species, including human. Exposure of the testis to body temperature (physiologic heating) [4], [8] or above (supraphysiologic heating) [9]–[12] results in increased germ cell degeneration, death and loss. Previous studies show that apoptosis, a process of programmed cell death, is a potential mechanism responsible for the germ cell death and loss induced by both physiologic heating [4] and supraphysiologic heating [10]–[13].

Recently, it was reported that autophagy could be activated in cells under varied stress conditions, such as ischemia injury [14], oxidative stress [15], endoplasmic reticulum stress [16], growth factor deprivation [17], heat stress [18], etc. Heat can induce an increase of autophagy in rat hepatocytes [18]. Autophagy is a process of self-degradation whereby cellular organelles and proteins are phagocytosed during metabolic stress [19]. It is an evolutionarily conserved physiological process that is thought to promote cell survival because nucleotides, amino acids, and free fatty acids can be generated during the degradation of cellular contents and then recycled and reused for macromolecular synthesis and ATP generation [20]. On the other hand, autophagy has also been shown to promote cell death under certain conditions. Apoptosis is not the sole means by which the cell can undergo a genetically programmed death. Autophagy has also been linked to the actual death process [21]. In fact, autophagy is often referred to as type II programmed cell death (distinct from type I programmed cell death, apoptosis) because it does not require caspase activation or DNA fragmentation, which are classical characteristics of apoptosis [20]. During the process of cell death, cross-talk between autophagy and apoptosis is very complex and still controversial. Different types of interplay (partnership or antagonist) between the autophagy and apoptosis have been indicated [21]–[23]. At present, the molecular mechanisms of autophagic process are still not fully understood. However, genetic studies in yeast have identified a set of autophagy-related genes (Atg genes) that are required for initiation and performance of autophagy.

In the protein profile of mouse spermatogenic cells that we previously established [24], [25], several homologous proteins of yeast ATG proteins have been identified. These results indicate that autophagy may play a role during spermatogenesis. In this study, the status of autophagy in germ cells after heat treatment, as well as the partnership between autophagy and apoptosis in these cells was investigated both *in vivo* and *in vitro* by using animal experiment and isolated spermatogenic cell line, respectively. The results indicate that autophagy and apoptosis act as partners in a cooperative manner to induce germ cell death after heat stress, and autophagy may do so via influence on the apoptotic pathway.

## Materials and Methods

### Animal and Cell Line

Young adult (7-wk-old) male ICR mice from the animal center of Nanjing Medical University (Nanjing, Jiangsu, China) were used and maintained under a controlled environment of 20–22°C, 12/12 h light/dark cycle, and 50–70% humidity, with food and water *ad libitum*. All experiments requiring the use of animals were approved by the Committee on the Ethics of Animal Experiments of Nanjng Medical University.

GC2-spd cells (immortalized mouse spermatocyte cell line which was established by stable cotransfection of freshly isolated mouse spermatocytes with the SV40 large T antigen gene and a temperature sensitive mutant of the p53 tumor suppressor gene, purchased from ATCC, Manassas, VA, USA) were used in this study, which were cultured in Dulbecco's modified Eagle's medium (DMEM) (Gibco BRL, Grand Island, NY, USA ) with 10% calf serum at 37°C and 5% CO_2_.

### Heat Treatment in Mice and GC2-spd Cells

The experiment of heat treatment on the mice testes was performed three times, and total eighteen mice were used for study. In each heating experiment, six mice were divided into six groups (untreated and 0.5 h, 2 h, 6 h, 12 h, 2 d after heat exposure). The untreated mouse was used as control, and the remaining mice were heat-treated mice. After anaesthesia with an intraperitoneal injection of sodium pentobarbital (40 mg/kg body weight), the tails and the scrotums containing the testes of heat-treated mice were immersed in a thermostatically controlled water bath at 42°C for 15 min, as previously described [10], [26]. Then we sacrificed these animals at 0.5 h, 2 h, 6 h, 12 h and 2 d after heat exposure. The testis tissue of each mouse was divided into 3 portions: one was fixed in Bouin’s solution and used for morphological examination, another fixed in 2% OsO4 for the ultrastructural examination, and the other was frozen in liquid nitrogen and prepared for the Western blot analyses.

The experiment of heat treatment on GC2-spd cells was performed three times. In each experiment, the cells were divided into comtrol group and heat-treated group. Untreated GC2-spd cells (two groups in each experiment) were used as control. The method of heat treatment on GC2-spd cells refer to our previous study [26]. In brief, GC2-spd cells were placed in a 42°C incubator for heating 3 h and then returned to incubate in a 37°C incubator. At 0 h, 3 h, 12 h and 24 h after heat exposure, the cells were collected for detection (two groups per time point in each experiment).

### Western Blotting

Samples containing 150 ug of protein from normal and heat-treated mouse testes or GC2-spd cells were electrophoresed on 12% sodium dodecyl sulfate **(**SDS) polyacrylamide gels and transferred to nitrocellulose membranes (Amersham Biosciences AB, Uppsala, Sweden). The membranes were blocked in phosphate-buffered saline (PBS) containing 5% nonfat milk powder for 1 h and then incubated overnight with a 1∶100-diluted anti-Atg7 rabbit polyclonal antibodies (Abcam, Cambridge, MA, USA) or a 1∶2000-diluted anti-LC3 (microtubule-associated protein light chain 3) rabbit polyclonal antibody (Novus Biologicals, Littleton, CO, USA) in PBS containing 5% nonfat milk powder. They were washed three times (10 min each) with PBS. The filters were then incubated for 1 h with horseradish peroxidase (HRP)-conjugated anti-rabbit IgG (Beijing ZhongShan Biotechnology CO., LTD, Beijing, China). Specific proteins were detected using an ECL kit (Amersham Biosciences, Buckinghamshire, England) and AlphaImager (FluorChem5500, Alpha Innotech, San Leandro, CA, USA). The protein bands obtained from western blot were analyzed by AlphaEaseFC software (Alpha Innotech, San Leandro, CA, USA) to determine the band density. The density of Atg7 band or LC3 band divided by the density of tubulin band, which represented the expression level of Atg7 or LC3. The ratio of LC3-II level/LC3-I level was used as a indicator of autophagic level.

### Transmission Electron Microscopy

For ultrastructural examination, testes from untreated mice (3 separate mice) and 12 hr after heat-treated mice (3 mice from 3 separate heating experiments, respectively) were postfixed with 2% OsO4 and embedded in Araldite. Ultrathin sections were stained with uranyl acetate and lead citrate and inspected using an electron microscope (JEM.1010; JEOL, Tokyo, Japan).

### Immunohistochemistry

Bouin’s solution-fixed paraffin-embedded sections from the mouse testis tissue were immunostained as described previously [27]. In brief, after quenching the endogenous peroxidase activity, the sections were blocked using a blocking serum and then incubated overnight at 4°C with primary antibodies to Atg7 (1∶200; Abcam). The sections were then incubated with HRP-conjugated secondary antibody (Beijing ZhongShan Biotechnology, Beijing, China). Immunoreactive sites were visualized brown with diaminobenzidine and mounted for bright field microscopy (Axioskop 2 plus; Zeiss, Germany). To confirm the specificity of Atg7 antibody, negative controls were processed in an identical manner, except that the primary antibody was replaced by normal IgG.

### RT-PCR

The primer sequences and the expected sizes of PCR products were as follows: Atg3: (sense) 5′-GGGAAAGGCTCTGGAAGT-3′ and (antisense) 5′-ATGTTGGACAGTGGTGGA-3′ (131 bp),; Atg5: (sense) 5′-CAGACAGAACCCGACCGA-3′ and (antisense) 5′-GTGAGCCTCAACCGCATC-3′ (493 bp); Atg7: (sense) 5′-CCTGCCCTACTTCTTATTCA-3′ and (antisense) 5′-TGCCTCCTTTCTGGTTCT-3′ (350 bp); Atg8: (sense) 5′-ACCAAGCCTTCTTCCTCC-3′ and (antisense) 5′-GCTGTCCCGAATGTCTCC-3′ (139 bp); Atg10: (sense) 5′-TGGCTGGGAATGGAGAAC-3′ and (antisense) 5′-CCTGCTGGGTGATGGTAT-3′ (359 bp); Atg12: (sense) 5′-CCTCGGAACAGTTGTTTATT-3′ and (antisense) 5′-CAGGACCAGTTTACCATCAC-3′ (107 bp); Atg16: (sense) 5′-GACATGATGGTGCGTGGAA-3′ and (antisense) 5′-CTGCATTGAGGCGATTGG-3′ (408 bp); β–actin: (sense) 5′-CCGTAAAGACCTCTATGCC-3′ and (antisense) 5′-CTCAGTAACAGTCCGCCTA-3′. Total RNA was extracted from the normal mouse testis using the Trizol reagent (Gibco BRL, Grand Island, NY, USA) and reverse-transcribed into cDNA with avian myeloblastosis virus (AMV) reverse transcriptase (Promega Corporation, Madison, WI, USA). PCR was performed using the following thermal cycling conditions: 5 min at 94°C; followed by 35 cycles of 30 sec at 94°C, 30 sec at 58°C–60°C and 30 sec at 72°C. β–actin was amplified in parallel as a positive control indicating the testis cDNA usable.

### Intratesticular Injection of Atg7 Small Interfering RNA (siRNA)

siRNAs against Atg7 mRNA (Invitrogen, Carlsbad, CA, USA. Cat. MSS232487, MSS232488, MSS292731) were purchased, diluted to a final concentration of 20 *µ*M, and stored at −20°C. The efficacies of the three siRNAs were verified using GC2-spd cells as per the manufacturer’s instructions, and the one with the highest efficacy was used for in vivo studies. Approximately 3–5 *µ*L of individual siRNA was injected into the seminiferous tubules of unilateral testis of each 7-week-old mouse using the injection procedure described previously [28]. The other testis of the same mouse was injected with Neg Control siRNA (Invitrogen, Carlsbad, CA, USA. Cat. 12935-200). Trypan blue (0.4%) and hoechst (10 µg/ml) were injected mixing with siRNA, used as indicators to ensure the microinjection successful. After injection, the testes were put back and the incisions were sutured. 48 after injection, the testis were heated as described previously. The mice were sacrificed at 12 h after heat exposure and testes tissues were obtained for study.

Total twelve mice were used in this experiment, six mice were used as untreated mice and the other six were heat-treated mice (48 after injection, the testis were heated). In the six mice of each group, the testes of three mice were used for extracting proteins to examine the expression of LC3, the testes from three other mice were used for TUNEL assay to indicate the cell apoptosis. The results from three separate mice were used for the statistics analysis.

### TUNEL Assay

The TUNEL assay for apoptotic cell detection was performed using the In Situ Cell Death Detection Kit (Boehringer Mannheim GmbH, Mannheim, Germany) according to the standard protocol described in the description. 18 testes tissues from 18 separate mice were used for TUNEL analysis. These 18 mice were divided into six groups (untreated control and 0.5 h, 2 h, 6 h, 12 h, 2 d after heat exposure) with 3 mice per group. The numbers of TUNEL-positive spermatogenic cells in approximately 250 seminiferous tubules of each mouse were counted, and the apoptotic indices were then determined by calculating the ratio of total numbers of TUNEL-positive cells/numbers of counted seminiferous tubules.

### Stable Transfection of the GC2-spd Cells with Atg7-targeted shRNA Vector

Mouse Atg7-targeted shRNA plasmid expression vector (Cat. MSH035820-2-CU6, MSH035820-3-CU6, MSH035820-4-CU6) and control plasmid expression vector (Cat. CSHCTR001-CU6) were purchased from GeneCopoeia Inc. (Rockville, MD, USA). The backbone of Atg7-targeted shRNA vector is psi-U6, and the hairpin loop sequence is TCAAGAG. The shRNA target sequences of MSH035820-2-CU6, MSH035820-3-CU6 and MSH035820-4-CU6 are ‘GACGTGACAVATAGCATCA’, ‘GGAGCAGCTCATTGATAAC’ and ‘TGGCTTCCTACTGTTATTG’, respectively.

Twenty-four hours before transfection, GC2-spd cells were incubated at 37°C with fresh DMEM medium without antibiotics. The subconfluent (80–90%) GC2-spd cells were then transfected with vectors using Lipofectamine™ 2000 (Invitrogen Corporation, Carlsbad, California, CA, USA) according to the instruction manual of Lipofectamine™ 2000. After antibiotic selection (3 µg/mL puromycin; Sigma-Aldrich, St. Louis, MO, USA), transfectants were pooled to avoid the effects of clonal selection and were expanded in 1.5 µg/mL puromycin. Western blot analysis was then performed to detect the expression of Atg7 protein.

### Apoptosis Detection by Flow Cytometry

Untreated cells (GC2-spd cells transfected with Atg7-targeted shRNA vector or GC2-spd cells transfected with the control vector) and cells at different times after incubation at 42°C, were collected for apoptosis detection. The detection of apoptosis by flow cytometry was performed according to the instruction manual of the FITC Annexin V Apoptosis Detection Kit I (BD Biosciences, San Diego, CA, USA). Briefly, 5×10^5^ cells were collected and washed twice with cold PBS. Cells were then resuspended in 500 µl Annexin V binding buffer and 5 µl of Annexin V-FITC and 5 µl of PI were added. The cells were gently vortexed and incubated for 10 min at room temperature in the dark. Within one hour, the cells were analyzed by FACS Calibur flow cytometer (BD, San Jose, CA, USA) using Cell Quest software (BD). Annexin V staining was analyzed in FL1, and PI staining was analyzed in FL2.

### Statistics Analysis

Data were analyzed by t-test for significant differences between the two groups. All the ratios were arcsine square root transformed before t-test analysis and the least significant difference (LSD) post hoc test was used to examine any significant difference between groups. The results were considered statistically significant when p<0.05.

## Results

### Heat Induced Apoptosis and Autophagy in the Germ Cells

We detected the status of apoptosis and autophagy in germ cells after heat treatment both *iv vivo* (animal experiment) and *in vitro* (isolated GC2-spd cell experiment).

**Figure 1:**
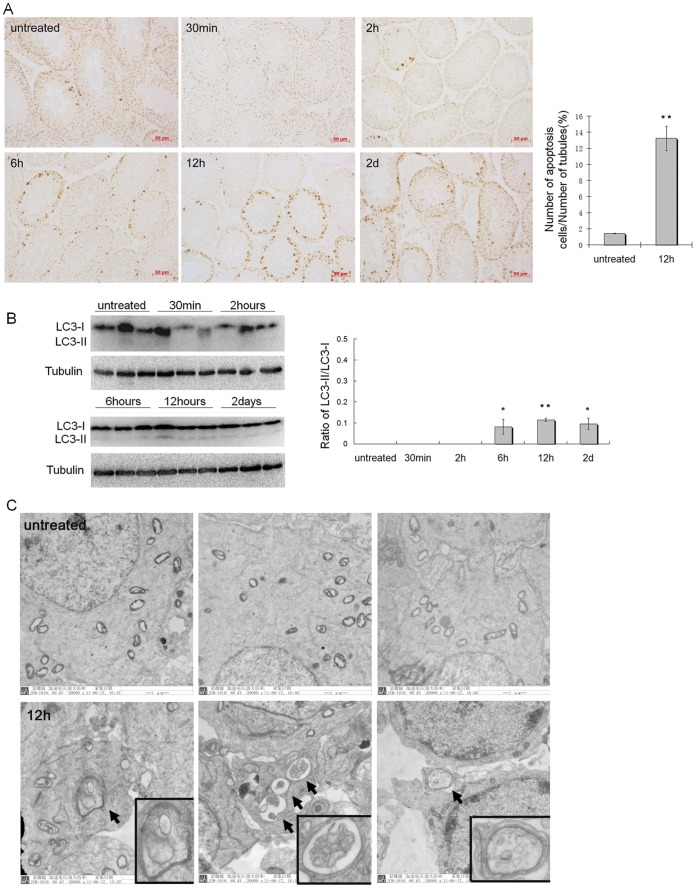
Heat induced apoptosis and autophagy in germ cells of mice testes. (A) Visualization of TUNEL positive apoptotic cells in sections of testis in untreated control mice and 30 min, 2 h, 6 h, 12 h, 2 d after heat-treated mice. Scale bar 50 μm; Right panel, apoptotic rate of spermatogenic cells in the control mice and mice at 12 h after heat treatment were presented in the bar charts (n = 3), **compared with untreated mice P<0.01. (B) Western blot for LC3 using lysates from testis of untreated mice or mice at different times after heat treatment; Right panel, the ratio of LC3-II/LC3-I were presented in the bar charts (n = 3), * compared with untreated mice P<0.05; ** compared with untreated mice P<0.01 (C) High resolution electron micrographs of testes tissues from untreated mice (n = 3, upper panel) and 12 h after heat-treated mice (n = 3, under panel). The arrows indicated the double membrane-surrounded autophagosomes in the spermatogenic cells.

*in vivo* - In the experiments of heat treatment on the mice testes, TUNEL assay was used for detecting cell apoptosis. As a result, the TUNEL-positive signals were mainly observed in the spermatocytes, and occasionally in the spermatogonia and round spermatids of the heat-treated testes (Fig. 1A). The numbers of TUNEL-positive apoptotic cells in the testes increased from 0.5 h after heat treatment when compared with that in the untreated testes, and the increase was significant at 12 h after heat treatment (P<0.01, Fig. 1A). The level of autophagy was assessed by detecting the conversion of LC3-I to LC3-II, which is a necessary step during the process of autophagy. LC3 is a mammalian homologus protein of yeast Atg8 protein, and the amount of LC3-II is known to correlate well with the number of autophagosomes [29], [30]. Our results showed that the conversion from LC3-I to LC3-II, demonstrated by the ratio of LC3-II/LC3-I, was significantly increased in mice testes at 6 h, 12 h and 2 d after heat treatment (Fig. 1B), and autophagosome was more frequently observed in the spermatogenic cells of heat-treated testes (Fig. 1C) when compared with the control testes. Above results suggested that heat could induce both apoptosis and autophagy in the testes of mice.

**Figure 2:**
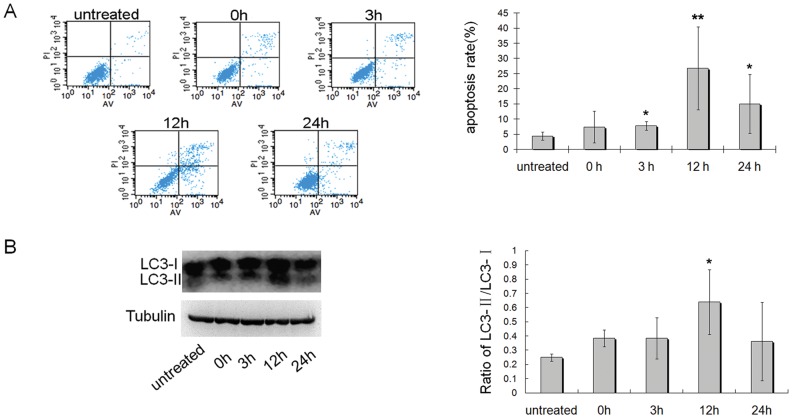
Heat induced apoptosis and autophagy in GC2-spd cells. (A) Untreated GC2-spd cells or GC2-spd cells at 0 h, 3 h, 12 h, 24 h after heat treatment were stained with Annexin V/PI and analyzed by flow cytometry to determine the apoptotic rate; Right panel, the apoptotic rate of GC2-spd cells were presented in the bar charts (n = 6), *compared with untreated cells P<0.05; **compared with untreated cells P<0.01. (B) Western blot for LC3 using lysates from untreated GC2-spd cells or cells at different times after heat treatment. Right panel, the ratio of LC3-II/LC3-I were presented in the bar charts (n = 3), *compared with untreated cells P<0.05.

*in vitro* - In the experiments of heat treatment on GC2-spd cells, we found the apoptotic rate of GC2-spd cells increased significantly at 3 h, 12 h and 24 h after heat incubation as compared with that of untreated cells (Fig. 2A). The apoptotic rate reached peak at 12 h and began to decrease at 24 h after heat. In our study the heat treatment on GC2-spd cells is transient and not all cells are induced to apoptosis by heat. The apoptotic cells may have died and been lost during the recovery period after heat treatment, whereas those unaffected cells can proliferate during this period. These may be the reason why there is a decline in apoptotic rate at 24 h after heat treatment. In addition, the conversion from LC3-I to LC3-II also was detected in the GC2-spd cells after heat incubation to assess the autophagy level of cells. The results showed the ratio of LC3-II/LC3-I was significantly increased in GC2-spd cells at 12 h after heat treatment (P<0.05, Fig. 2B). These results suggested that heat induced an increase of apoptosis and autophagy in GC2-spd cells.

### Ubiquitin-like Protein Conjugation Pathways in the Mouse Testis

**Figure 3:**
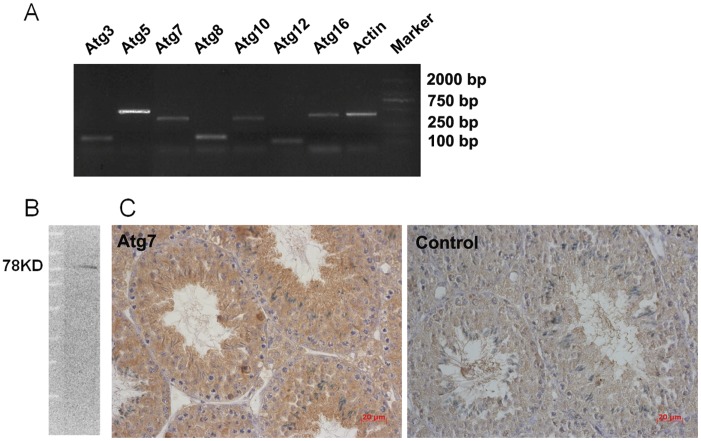
Ubiquitin-like protein conjugation pathways exist in the mouse testis. (A) The mRNA expression of Atg3, Atg5, Atg7, Atg8, Atg10, Atg12, Atg16 in mice testes (n = 3). (B) Western blot analysis was performed using anti-Atg7 polyclonal antibodies for total protein extracts prepared from normal mouse testis (n = 3). (C) Immunohistochemical staining of the mouse testis using anti-Atg7 polyclonal antibodies and normal IgG (control) (n = 3). The results showed Atg7 protein was expressed in Leydig cells, Sertoli cells and all types of spermatogenic cells. Scale bar 20 μm.

Atg12-Atg5 and Atg8 (LC3)-PE (phosphatidylethanolamine) systems constitute an ubiquitin-like protein conjugation pathway, which mediated the formation of autophagosomes. Several ATG genes, including Atg3, Atg5, Atg7, Atg8, Atg10, Atg12 and Atg16, participate in this pathway [31], [32]. By RT-PCR, we confirmed on the mRNA level that all of these ATG genes were expressed in mice testes (Fig. 3A). Atg7 is an upstream protein in the ubiquitin-like protein conjugation pathway and it is an essential factor for the formation of autophagosome [31], [33]. We further confirmed the expression of Atg7 in the mouse testes on the protein level by using Western blotting (Fig. 3B), and immunohistochemical results showed that Atg7 protein was expressed in Leydig cells, Sertoli cells and all types of spermatogenic cells (Fig. 3C). The above results confirmed that the ubiquitin-like protein conjugation pathways existed in the testis.

### Expression Level of Atg7 Protein Related to Apoptosis and Autophagy of Germ Cells

To confirm Atg7 protein and involved ubiquitin-like conjugation systems are responsible for heat-induced autophagy and then participated in the impairment of spermatogenesis, the relative expression of Atg7 with the level of apoptosis or autophagy was examined in germ cells after introducing heat both *iv vivo* and *in vitro*.

**Figure 4:**
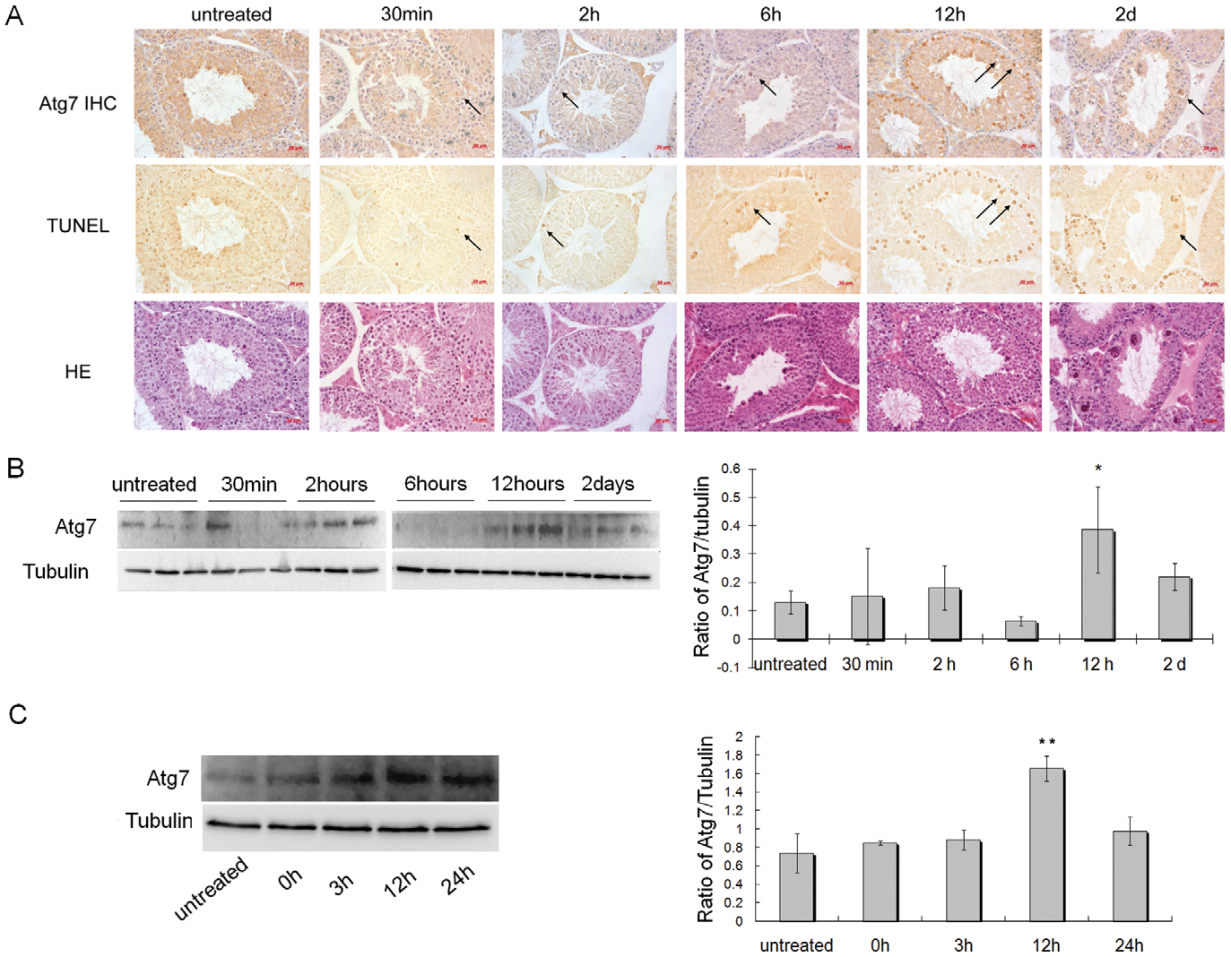
Expression level of Atg7 protein related to apoptosis and autophagy in germ cells. (A) The serial sections of testes from untreated (n = 3) or heat-treated mice (n = 3) were used for immunohistochemical detection of Atg7, TUNEL staining and HE staining. The TUNEL-positive apoptotic cells (mainly were spermatocytes) and the cells with increased Atg7 expression were observed in colocalization (arrow). Scale bar 20 μm. (B) The expression level of Atg7 protein in the mouse testis at different times after heat incubation or without heat treatment; Right panel, densitometric quantitation of Atg7 expression were presented in the bar charts (n = 3), *compared with untreated mice P<0.05. (C) The expression level of Atg7 protein in the GC2-spd cells at different times after heat incubation or without heat treatment. Right panel, densitometric quantitation of Atg7 expression were presented in the bar charts (n = 3), **compared with untreated cells P<0.01.

*in vivo* - The serial sections of mice testes after heat treatment were used for immunohistochemical examination to detect the expression of Atg7 protein, for TUNEL staining to detect apoptotic cells, and for HE staining to examine the testis structure. The results showed that the number of TUNEL-positive apoptotic cells (which were mainly spermatocytes) increased markedly in the mouse testis after heat treatment when compared with that of untreated mice, which was in accord with the expression characteristic of Atg7 protein in the heat-treated testes. The increased expression of Atg7 protein in many spermatocytes and occasionally in spermatogonia and round spermatids was observed in the testes after heat treatment. Most of these spermatocytes with increased expression of Atg7 were the TUNEL-positive apoptotic cells (Fig. 4A). Results of Western Blot also showed that the expression of Atg7 in the testes increased significantly after heat treatment (Fig. 4B), which coincided with the increased levels of apoptosis and autophagy (Fig. 1A and 1B). Above results indicated that Atg7 protein might participate in regulating autophagy and apoptosis in the germ cells of mice testes after heat.

*in vitro* - We examined the relative expression of Atg7 with apoptosis and autophagy in GC2-spd cells after heat exposure. The expression of Atg7 also increased in heat-treated GC2-spd cells (Fig. 4C), which coincided with the levels of apoptosis and autophagy that was described previously (Fig. 2A and 2B). The above results indicated that Atg7 protein might participate in regulating autophagy and apoptosis in GC2-spd cells after heat.

### Atg7 Knockdown Conferred Significant Protection against Heat-induced Autophagy in Germ Cells

We knockdown the expression of Atg7 *in vivo* and *in vitro* to study the role of Atg7 on heat-induced autophagy in germ cells.

**Figure 5:**
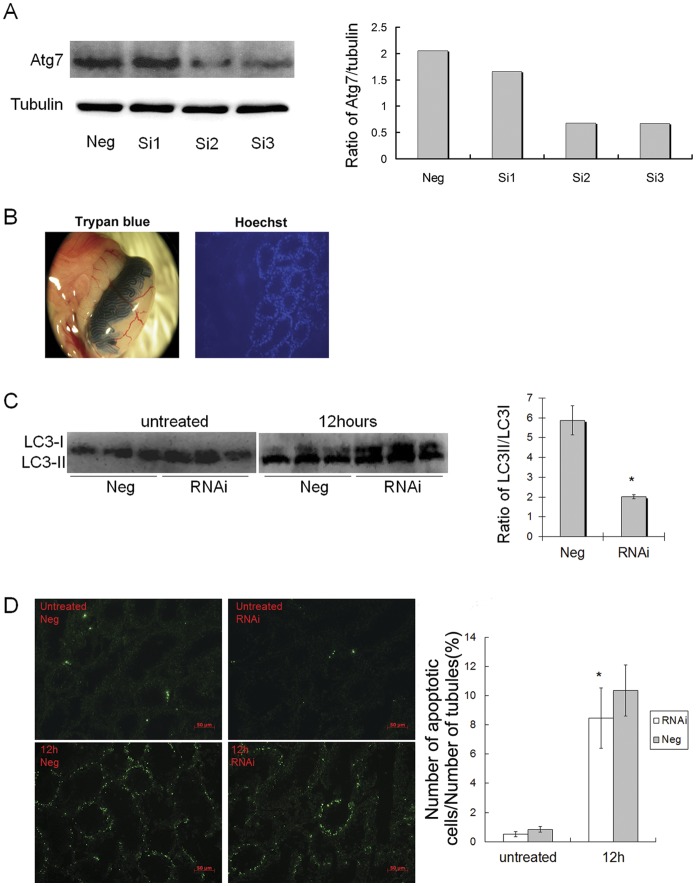
Atg7 knockdown inhibited heat-induced autophagy and apoptosis in testes of mice. (A) Western blot analysis showed that the Atg7 protein expression was markedly repressed in the Atg7-targeting siRNA cells; Right panel, the efficacies of three Atg7-targeting siRNAs were presented in the bar charts. Si1: MSS232487; Si2: MSS232488; Si3: MSS292731; Neg: Neg control siRNA. (B) Intratesticular injection of Atg7 small interfering RNA (siRNA) showing about 40% of the seminiferous tubules were successfully introduced with siRNA materials (left), and siRNA material could be observed in all types of spermatogenic cells in the seminiferous tubule (right). (C) Only LC3-I band could be detected both in the Atg7-targeting siRNA and the control testes before heat treatment. At 12 h after heat treatment, both the LC3-I and LC3-II bands were observed, and Atg7-targeting siRNA testis exhibited a marked decrease in the rate of conversion of LC3 compared to Neg control testis. Right panel, the ratio of LC3-II/LC3-I in 12 h after heat-treated testes were presented in the bar charts (n = 3), *compared with Neg control testis P<0.01. (D) At 12 h after heat treatment, Atg7-targeting siRNA testis exhibited a marked decrease in the rate of cell apoptosis compared to Neg control testis. Scale bar 50 μm; Right panel, the apoptotic rate of spermatogenic cells were presented in the bar charts(n = 3), *compared with Neg control testis P<0.05.

*in vivo* - Mice testes were injected with Atg7-targeting siRNA to down-regulate Atg7 expression and the subsequent effects on protein expression in correlation to the level of autophagy in the testes were observed. By examining the efficacies of three Atg7 targeted siRNAs using GC2-spd cells, the one with the highest efficacy (Cat. MSS292731) was selected for injection (Fig. 5A). The seminiferous tubules of unilateral testis in 7 weeks old mice were injected by rete injection with this Atg7-targeted siRNAs. Trypan blue and hoechst also were injected mixing with the siRNA, which could be used as indicators to confirm the successful microinjection. By observing the area staining with trypan blue in the testis, we found the mixture of trypan blue, hoechst and siRNA could be penetrated into about 40% seminiferous tubule. In addition, hoechst could be observed in all sorts of spermatogenic cells of the seminiferous tubule under the fluorescence microscope, which indicated the mixture including siRNA penetrated into these cells (Fig. 5B). The other testis of mice was injected with negative control siRNA. Two days after the injection, the testes were treated with heat. Western blot analysis showed only LC3-I band could be detected both in the Atg7-targeted siRNA and the control testes before heat treatment, which indicated the conversion of LC3 was very weak in the untreated testes (Fig. 5C). At 12 h after heat treatment, both the LC3-I and LC3-II bands were observed, and the ratio of LC3-II/LC3-I decreased significantly in the Atg7-targeted siRNA testes as compared with the Neg control testes (P<0.01; Fig. 5C). These results indicated that Atg7 knockdown decreased the heat-induced autophagy in the mice testes.

**Figure 6:**
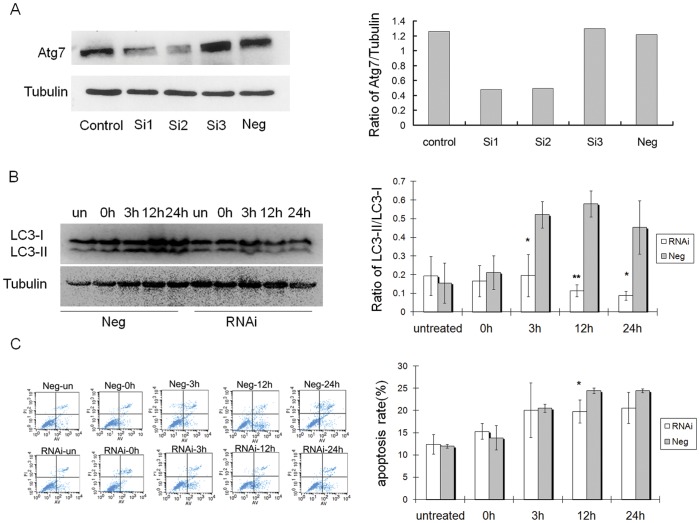
Atg7 knockdown inhibited heat-induced autophagy and apoptosis in GC2-spd cells. (A) Western blot analysis showed the expression of Atg7 protein was markedly repressed in the Atg7-targeting siRNA (MSH035820-2-CU6) cells when compared with the control cells. Right panel, the efficacies of three Atg7 targeting shRNA vector were presented in the bar charts. control: untransfected cells; Si1: MSH035820-2-CU6, Si2: MSH035820-3-CU6, Si3; MSH035820-4-CU6. Neg: CSHCTR001-CU6. (B) Three hours after cell warming, the rate of conversion of LC3 became decreased in Atg7-targeting siRNA cells and was significantly different as compared with Neg control cells; Right panel, the ratio of LC3-II/LC3-I were presented in the bar charts (n = 3), *compared with Neg control cells P<0.05; **compared with Neg control cells P<0.01. (C) The apoptotic rates increased both in Atg7-targeting siRNA cells and Neg control cells after cell warming, but the apoptotic rate of Atg7-targeting siRNA cells was lower than that of control cells, and the difference was siginificant at 12 h after heat stress; Right panel, the apoptotic rate of GC2-spd cells were presented in the bar charts (n = 3), *compared with Neg control cells P<0.05.

*in vitro* - The expression of Atg7 was also down-regulated by transfecting the Atg7-targeted shRNA vector in GC2-spd cells. It was found that the expression of Atg7-targeted shRNA (Cat. MSH035820-2-CU6) resulted in a obvious decrease in Atg7 protein levels, as detected by Western blot (Fig. 6A). At 3 h, 12 h and 24 h after cell warming (incubation at 42°C for 3 h), the ratio of LC3-II/LC3-I was decreased in Atg7-targeted siRNA cells which showed a significant difference as compared with Neg control cells (Fig. 6B). The above results indicated that Atg7 knockdown decreased the heat-induced autophagy in GC2-spd cells.

### Autophagy was not a Protective Mechanism against Heat-induced Apoptosis

When autophagy was repressed by down-expression of Atg7 in germ cells *in vivo* and *in vitro*, a decreased rate of apoptosis was detected.

*in vivo* - When the autophagy was down-regulated in the 12 h after heat-treated testes by suppressing the Atg7 expression using siRNA injection (Fig. 5C), it was found that the apoptotic rate of spermatogenic cells in the testes at this time also decreased markedly as compared with the control testes (P<0.05; Fig. 5D, Table S1). The above results indicated that autophagy might not be a protective mechanism against heat-induced apoptosis, but play a positive role in the process of germ cells apoptosis.

*in vitro* - After heat-induced autophagy was markedly suppressed by Atg7-targeted siRNA in GC2-spd cells (Fig. 6B), cell apoptosis was still detected. It was found that the apoptotic rates increased both in Atg7-targeted siRNA cells and Neg control cells after cell warming, but the apoptotic rate of Atg7-targeted siRNA cells was lower than that of control cells, and the difference was siginificant at 12 h after heat stress (P<0.05; Fig. 6C, Table S2).

## Discussion

The overall goal of this study is to clarify the role of autophagy in the impairment of spermatogenesis after heat treatment, by which we can further understand the molecular mechanism of spermatogenesis and might indentify new targets for infertility diagnosis and male contraceptive development.

Testicular heating can disrupt spermatogenesis and cause subfertility [13], [33], [34]. Clinically, cryptorchidism is a typical case of testicular heating in which the testes are exposed to body temperature rather than scrotal temperature, and abnormal testis function and damaged spermatogenesis are routinely observed in these cases [35], [36]. In addition, a supraphysiologic heating (higher than body temperature) on the testis also can disrupt spermatogenesis [12], [26]. It has been indicated that histological changes in the testes after exposure to body temperature (physiologic heating) or above (supraphysiologic heating) are marked by germ cell loss via apoptotic pathways, and germ cell apoptosis might be an potential mechanism responsible for the impairment of spermatogenesis induced by heat [4], [10]–[13], [37]. In this study, heat (42°C) induced increase in apoptosis of germ cells was also confirmed by *in vivo* and *in vitro* experiments (Fig. 1A and 2A).

In recent years, heat (41°C for 0.5 hr) induced autophagy has been reported in somatic cells [18]. Autophagy, which has been shown to promote cell death under certain conditions, is now often referred to as type II programmed cell death (distinct from type I programmed cell death, apoptosis). It has been reported that cross-talk between apoptosis and autophagy is complex in the sense that they might act synergistically or antagonistically with each other in the process of cell life and death [20], [21]. In this study we found that testicular heating not only induces apoptosis, but also induces autophagy in germ cells (Fig. 1B, 1C and 2B). These results imply that autophagy might be another potential mechanism participating in germ cell death after heat stress. It should be noted that the temperature achieved and the duration of heating on testes are parameters that have a influence of the effects observed on spermatogenesis, and the cell changes in the testes are not identical under different heating conditions [37]–[39]. For example, it was reported that blood flow through the testes could be elevated after a heat of 43–45°C on the testes, but not affected by the exposure of testes to body temperature, and the changed blood flow could influence the testis cells [39]. In our study only one heating condition was used. So the cell changes that we have observed in the testes may not represent the cell response in all cases of testicular heating.

Molecular mechanisms for autophagy induction in mammalian cells are still not fully understood but at least 30 autophagy-related genes (Atg genes) have been identified in yeast [32]. These include genes that regulate autophagosome formation, which requires two evolutionarily conserved ubiquitin-like conjugation systems–the Atg12-Atg5 and the Atg8 (LC3)-PE (phosphatidylethanolamine) systems [32]. Atg genes were originally described in yeast, and in some cases their orthologs have been isolated and functionally characterized in mammals [40]. In this study, the expression of Atg12-Atg5 and Atg8 (LC3)-PE systems in mouse testis was demonstrated by RT-PCR, and the expression of Atg7, a gene upstream to both these two systems, was further confirmed in the testis at the protein level. These results confirm that the ubiquitin-like conjugation systems exist in the testis, and may be responsible for heat-induced autophagy in germ cells.

Atg7 is required for initiating the formation of autophagic vacuoles [41], and the down-expression of Atg7 in mouse L929 cells reduced autophagic cell death [42]. In this study, it was demonstrated that Atg7 was expressed in all types of spermatogenic cells, and its expression level positively correlated with the level of autophagy in germ cells (Fig. 1B and 4B, 2B and 4C). Thus, Atg7 was selected as the investigative target to represent the level of autophagy involving the ubiquitin-like conjugation pathway, as to further analyze the role of autophagy in heat-induced germ cell death. This study showed that down-expression of Atg7 protein indeed resulted in notable decrease of autophagic level in germ cells after heat stress, both *in vivo* and *in vitro*, and this down-regulation of autophagy further reduced the cell apoptosis significantly (Fig. 5 and 6). Previous studies have reported that autophagy can act as a partner or an antagonist to apoptosis during cell death. When autophagy and apoptosis act as partners to induce cell death, they may do so independently via parallel pathways, or one may influence the other [21]. In this study, our results showed that down-regulation of autophagy can decrease the cell apoptosis, but not increase the cell apoptosis. These results suggest autophagy and apoptosis act as partners, not antagonist, to induce cell death in the testes after heat. Furthermore, autophagy may do so via influence the apoptotic pathway in a cooperative manner. If autophagy and apoptosis act as partners to induce cell death via parallel pathways, down-regulation of autophagy should not influence the apoptotic level.

In conclusion, the present study demonstrates that heat triggers autophagy and apoptosis in germ cells, which act as partners to induce cell death and lead to destruction of spermatogenesis. Atg7 and its attended ubiquitin-like conjugation systems are critically involved in the heat-mediated autophagy.

## Supporting Information

Table S1 **The raw data and the calculated p values for** **Fig. 5D** **indicated the decreased apoptotic rate of spermatogenic cells in Atg7-targeting siRNA testis.**
(DOC)

Table S2 **The raw data and the calculated p values for** **Fig. 6C** **indicated that apoptotic rate of Atg7-targeted siRNA cells was lower than that of Neg control cells.**
(DOC)
