# Supplementary material for: Autophagy and Apoptosis Act as Partners to Induce Germ Cell Death after Heat Stress in Mice
Source: PLoS One. 2012 Jul 25;7(7):e41412. doi: 10.1371/journal.pone.0041412 (PMC3405141; doi:10.1371/journal.pone.0041412)
Supplement: Table S1 — The raw data and the calculated p values for Fig. 5D indicated the decreased apoptotic rate of spermatogenic cells in Atg7-targeting siRNA testis. (DOC) [file pone.0041412.s001.doc]

|  | **Raw data**  (Number of apoptotic cells  /number of tubules, %) | | **Data were arcsine square**  **root transformed** | | |
| --- | --- | --- | --- | --- | --- |
|  | **RNAi** | **Neg** | **RNAi** | **Neg** | **P value** |
| **untreated** | 1.010 | 1.111 | 0.1007 | 0.1056 | 0.376 |
|  | 0.784 | 0.742 | 0.0887 | 0.0862 |  |
|  | 0.542 | 0.682 | 0.0737 | 0.0827 |  |
| **12h after**  **heat treatment** | 10.868 | 13.297 | 0.3359 | 0.3733 | 0.044 |
|  | 7.754 | 8.782 | 0.2822 | 0.3009 |  |
|  | 6.758 | 9.014 | 0.2630 | 0.3049 |  |

**Supplemental Table 1. The raw data and the calculated p values for Fig 5D indicated the decreased apoptotic rate of spermatogenic cells in Atg7-targeting siRNA testis.**

Paired t-test was used for statistics analysis in this experiment (data were compared between RNAi and Neg groups). RNAi: the rate of cell apoptosis in Atg7-targeting siRNA testis. Neg: the rate of cell apoptosis in Neg control testis.
