# Supplementary material for: Autophagy and Apoptosis Act as Partners to Induce Germ Cell Death after Heat Stress in Mice
Source: PLoS One. 2012 Jul 25;7(7):e41412. doi: 10.1371/journal.pone.0041412 (PMC3405141; doi:10.1371/journal.pone.0041412)
Supplement: Table S2 — The raw data and the calculated p values for Fig. 6C indicated that apoptotic rate of Atg7-targeted siRNA cells was lower than that of Neg control cells. (DOC) [file pone.0041412.s002.doc]

**Supplemental Table 2. The raw data and the calculated p values for Fig 6C indicated that** **apoptotic rate of Atg7-targeted siRNA cells was lower than that of Neg control cells.**

|  | **Raw data**  ( apoptotic rate, %) | | **Data were arcsine square**  **root transformed** | | |
| --- | --- | --- | --- | --- | --- |
|  | **RNAi** | **Neg** | **RNAi** | **Neg** | **P value** |
| **untreated** | 14.42 | 12.44 | 0.3895 | 0.3605 | 0.499 |
|  | 14.46 | 12.03 | 0.3901 | 0.3542 |  |
|  | 10.37 | 11.49 | 0.3279 | 0.3458 |  |
| **0h after**  **heat treatment** | 16.17 | 11.11 | 0.4138 | 0.3398 | 0.551 |
|  | 13.56 | 12.94 | 0.3771 | 0.3680 |  |
|  | 15.79 | 17.63 | 0.4086 | 0.4333 |  |
| **3h after**  **heat treatment** | 16.19 | 21.6 | 0.4141 | 0.4834 | 0.808 |
|  | 16.43 | 20.56 | 0.4174 | 0.4706 |  |
|  | 26.84 | 19.4 | 0.5446 | 0.4561 |  |
| **12h after**  **heat treatment** | 17.16 | 25.28 | 0.4271 | 0.5268 | 0.026 |
|  | 17.38 | 23.84 | 0.4300 | 0.5101 |  |
|  | 21.90 | 24.22 | 0.4870 | 0.5145 |  |
| **24h after**  **heat treatment** | 16.82 | 24.91 | 0.4226 | 0.5226 | 0.101 |
|  | 18.15 | 24.64 | 0.4401 | 0.5194 |  |
|  | 24.03 | 23.80 | 0.5123 | 0.5096 |  |

Unpaired t-test was used for statistics analysis in this experiment (data were compared between RNAi and Neg groups). RNAi: the apoptotic rate in Atg7-targeting siRNA cells. Neg: the apoptotic rate in Neg control cells.
